# Linking N_2_O Emission with AOB and *nirK*-Denitrifier in Paddy Fields of Karst and Non-Karst Areas

**DOI:** 10.3390/microorganisms13112633

**Published:** 2025-11-20

**Authors:** Zhenjiang Jin, Weijian Chen, Wu Yuan, Yunlong Sun, Xiaoyi Xiao, Heyao Liang, Chengxi Yang, Bin Dong

**Affiliations:** 1 College of Environmental Science and Engineering, Guilin University of Technology, Guilin 541006, China; 2 Guangxi Key Laboratory of Environmental Pollution Control Theory and Technology, Guilin University of Technology, Guilin 541006, China; 3 Guangxi University Engineering Research Center of Watershed Protection and Green Development, Guilin University of Technology, Guilin 541006, China; 4 Key Laboratory of Carbon Emission and Pollutant Collaborative Control, Education Department of Guangxi Zhuang Autonomous Region, Guilin University of Technology, Guilin 541006, China; 5 Guangxi Ecological and Environmental Protection Modern Industry College, Guilin University of Technology, Guilin 541006, China

**Keywords:** karst, paddy field, N_2_O, AOB, *nirK*-denitrifier, Ca^2+^, pH

## Abstract

Denitrification and nitrification are two pivotal microbial processes relating to N_2_O emissions. However, the difference in N_2_O emission fluxes and N_2_O-producing bacteria between a karst (KA) and non-karst area (NKA) remains unclear. The objective of this study is to compare the differences in soil N_2_O emissions, nitrifying bacteria, and denitrifying bacteria during the growth period of rice in KA and NKA, and to explore the mechanisms by which microorganisms and environmental factors drive N_2_O emissions. Here, N_2_O emission fluxes of paddy fields were collected using the static dark chamber and measured using gas chromatography at KA and NKA in the Maocun Karst Experimental Site in Guilin, China. The nitrifying bacteria (ammonia-oxidizing bacteria, AOB) and denitrifying bacteria (*nirK*-denitrifier) were determined using real-time PCR and high-throughput sequencing, respectively. Results showed that during the rice growth period, the N_2_O emission fluxes in KA was generally lower than that in NKA, with cumulative N_2_O emissions of −0.054 and 0.229 kg·hm^−2^ in KA and NKA, respectively. The absolute abundance of AOB in KA (8.91 × 10^6^–2.68 × 10^7^ copies·g^−1^) was significantly higher than that in NKA (1.57 × 10^6^–6.48 × 10^6^ copies·g^−1^), while the absolute abundance of *nirK*-denitrifier had no significant difference between the two areas. The composition and diversity of AOB and *nirK*-denitrifier differed significantly between KA and NKA. Results from partial least squares structural equation modeling (PLS-SEM) indicated that soil properties, carbon sources, and nitrogen sources had positive effects on AOB and *nirK*-denitrifier, while *nirK*-denitrifier had a negative effect on N_2_O emissions. Partial least squares regression (PLSR) predictions revealed that NO_3_^−^-N, SOC, TN, Mg^2+^, Ca^2+^, and pH were the most important factors influencing N_2_O emission fluxes. This study highlights the critical role of the typical characteristics of KA soils in reducing N_2_O emissions from paddy fields by driving the evolution of AOB and *nirK*-denitrifier.

## 1. Introduction

Nitrous oxide (N_2_O) is the third greenhouse gas (GHG) after carbon dioxide (CO_2_) and methane (CH_4_) [1,2]. Soils contribute about 60% of global N_2_O emissions [3], while agricultural soils account for nearly 84% of them [4]. Thus, studying the processes and mechanisms of N_2_O emissions from farmland soils is of great importance.

Rice agriculture is predominantly in China and Southeast-Asian countries, supporting over 60% of the global population [5]. Long-term flooded cultivation has formed a specific soil type—paddy soil [5,6]. However, increasing rice demand and extensive nitrogen (N) fertilizer application [7] have made rice paddy a major source of N_2_O emissions [8,9]. Therefore, investigating the processes and mechanisms of N_2_O emissions from paddy soils is crucial for mitigating global climate change.

Nitrification is one of the core processes contributing to N_2_O production [10,11,12,13]. The N-fertilizer and native N in soils provide available substrates for nitrification, initiating N mineralization [10,13] and thereby triggering N_2_O emissions [14]. Soil organic carbon (SOC) is a key soil component, which directly influences soil physicochemical properties such as pH [15], which are major factors determining the potential for N_2_O emissions [16]. In alkaline soils with high nitrification rates [12], N_2_O emissions decrease with increasing SOC [12] and pH [17]. For instance, in neutral paddy soils, biochar addition can reduce N_2_O emissions by increasing pH [18]. However, some studies report that cumulative N_2_O emissions increase significantly with rising pH under aerobic conditions [12]. In summary, the nitrification controlled by soil physicochemical properties (such as pH, SOC, and N content) is a key process in N_2_O emissions from agricultural soils, driven by the combined effects of N-fertilizer input and soil background N mineralization [19,20].

Ammonia-oxidizing bacteria (AOB) are important bacteria dominating the nitrification process [21,22] and are sensitively regulated by soil physicochemical properties. Studies show that AOB contribute to about 85% of nitrification-related N_2_O emissions, which vary with soil pH [23]. In flooded and alkaline paddy soils [24] with high N [13,25], oxygenated zones near rice roots promote nitrification primarily mediated by AOB due to rhizosphere respiration, leading to N_2_O emissions. Fertilizer-derived N_2_O emissions are positively correlated with AOB abundance and negatively correlated with soil pH [14]. In acidic paddy soils, AOB are the primary ammonia-oxidizing microorganisms in both abundance and function [26] with autotrophic nitrification being dominant [27]. At different depths of soil profiles, AOB abundance is positively correlated with N_2_O emissions [28]. Thus, alternating wet–dry conditions can change soil properties and oxygen availability, leading to variations in AOB abundance and community structure, thereby altering nitrification activity [26]. SOC, as a carbon and energy source for bacteria, also influences N_2_O emissions [20]. These findings indicate that soil physicochemical properties regulate N_2_O emissions during nitrification by influencing AOB community.

Denitrification and dissimilatory NO_3_^−^ reduction to NH_4_^+^ are the other core processes contributing to N_2_O emissions in agricultural soils [12,29], which is affected by environmental parameters, functional microbe composition, and key enzyme activities [19,20,29]. The flooded rice paddy creates anaerobic conditions that facilitate N_2_O emissions via denitrification [24]. Biochar addition significantly reduces N_2_O emissions from denitrification in acidic paddy soils [30]. Therefore, regulating N_2_O emissions from denitrification through exogenous amendments is important for optimizing N-cycle management.

Denitrifying bacteria are key microbial groups in the denitrification process [21,22]. In agricultural soils, fertilizer application [31] and straw return [32] significantly alter soil physicochemical properties, carbon, and nitrogen levels (e.g., pH and SOC), strongly influencing the growth, population structure, composition, and diversity of *nirS*- and *nirK*-denitrifier [33,34], thereby changing N_2_O emissions [14]. Flooded paddy fields host diverse bacteria adapted to anaerobic conditions, particularly *nirK*-denitrifier [35], which is influenced by the changes in soil environment, such as water regime and root growth, thereby altering denitrification and N_2_O emissions. Insufficient nutrients, for example dissolved organic carbon (DOC), could reduce *nirK*-denitrifier abundance, limit denitrification, and further decrease N_2_O emissions [36]. The activity, rather than the abundance of *nirK*-denitrifier is a key factor determining N_2_O emission potential [16], suggesting that their function is more important than their abundance.

In KA, intense dissolution of carbonates (mainly limestone and dolomite) results in soils with higher exchangeable Ca^2+^ and Mg^2+^ concentrations and pH [37,38]. SOC and total N (TN) in KA are also significantly higher [39,40], where Ca^2+^ and Mg^2+^ promote strong immobilization of organic matter, dissolved N, and phosphorus [39], changing soil N mineralization and nitrification [41] and potentially affecting N_2_O emissions. It has been reported that denitrification controls N_2_O emissions under high soil moisture conditions (70% water-filled pore space) in KA [20]. Thus, N_2_O emissions from paddy soils with variable moisture conditions (flooding early and drainage later) may fluctuate significantly in KA. In summary, the unique physicochemical environment of paddy fields in KA likely influences the N-cycle by suppressing N mineralization and nitrification, ultimately regulating N_2_O emissions.

Compared to NKA, the distinct soil properties of KA support unique microbial communities that have adapted over time to form stable assemblages. These microbe exhibit differences in C and nutrient utilization efficiency, leading to variations in microbial function [37]. In KA, vegetation drives the evolution of *nirK*-denitrifier by altering the rhizosphere environment [42], with dominant *nirK*-denitrifier such as *Bradyrhizobium* showing positive correlations with SOC and TN [43]. It has been reported that KA forest soils were inhabited significantly by more *nirK*-denitrifier such as Bacillales compared to NKA [39], while pH and nutrients are key factors influencing denitrification and N_2_O emissions. However, the coupling relationships between soil physicochemical properties, AOB/*nirK*-denitrifier, and N_2_O emissions during the rice growth period in KA and NKA remain unexplored.

Based on the above research, we hypothesize that significant differences in soil physicochemical factors (e.g., pH, SOC, and Ca^2+^) between KA and NKA drive notable changes in the community structure, composition, and diversity of AOB and *nirK*-denitrifier, thereby altering soil N_2_O emissions. To test this hypothesis, this study was conducted at the Maocun Karst Experimental Site in Guilin, China, including KA and NKA. The following investigations were carried out: (1) measurement of soil physicochemical properties; (2) determination of N_2_O emission fluxes and cumulative emissions; (3) analysis of the absolute and relative abundances of AOB and *nirK*-denitrifier communities; and (4) use of partial least squares structural equation modeling (PLS-SEM) and partial least squares regression (PLSR) to explore the underlying drivers of N_2_O emission fluxes and cumulative emissions in KA and NKA.

## 2. Materials and Methods

### 2.1. Study Area and Sampling Site Description

The study area is 30 km from the Guilin city, within a subtropical monsoon climate zone. The region comprises both KA and NKA geological formations. The paddy fields in KA with an area of 127 m^2^ (25°08′30″ N, 110°31′28″ E) and in NKA with an area of 140 m^2^ (25°10′51″ N, 110°31′35″ E) were selected as experimental sites. Each field was divided into three replicate plots with the same area (KA: 42.20 m^2^ and NKA: 46.67 m^2^) using PVC ridge. Each plot was subjected to uniform irrigation, fertilization, and management practices. The soil in KA is classified as limestone soil, while that in NKA is zonal silicate red soil. The rice growth cycle spanned 93 days for a single season. Prior to seedling transplantation, 7.56 kg (KA) and 8.53 kg (NKA) of compound fertilizer (N-P_2_O_5_-K_2_O, 18% each) was applied in each plot as basal fertilizer. On 14 July, an additional topdressing was applied at 5.70 kg (KA) and 6.40 kg (NKA) of the same compound fertilizer. The plot information can be found in reference [44].

### 2.2. Sample Collection and Preparation

To characterize variations in soil properties and communities of AOB and *nirK*-denitrifier during the rice growing, soil samples were collected on eight dates: 4 June (transplanting), 12 June (seedling), 26 June (tillering), 10 July (tillering), 24 July (jointing), 7 August (booting), 21 August (heading), and 4 September (maturity). Three adjacent soil samples of 0–20 cm in each plot were collected and thoroughly mixed and then transported to the laboratory quickly. Visible plant roots, debris, and other extraneous materials were removed from samples. All samples were divided into two subsamples: one was stored stored at −80 °C for microbial analysis, while the other was air-dried for the determination of physicochemical properties and nutrient content.

### 2.3. Analysis of Physicochemical Properties

Soil physicochemical properties were analyzed by routine methods [45,46]. Soil water content was determined using the oven-drying method. Soil pH was measured directly with a Leici PHS-3E pH meter (Shanghai Yidian Scientific Instrument Co., Ltd., Shanghai, China), using CO_2_-free distilled water as the extractant at a water-to-soil ratio of 2.5:1. Soil organic carbon (SOC) was analyzed by the potassium dichromate external heating method with concentrated sulfuric acid [47]. Dissolved organic carbon (DOC) was extracted using the water–soil oscillation method using an analyzer (Analytikjena C3100, Jena, Germany). Total nitrogen (TN) was determined by the Kjeldahl method with concentrated sulfuric acid digestion. Alkali-hydrolyzable nitrogen (AN) was measured using the alkali diffusion method. Ammonium nitrogen (NH_4_^+^-N) was analyzed by the Nessler’s reagent colorimetric method, and nitrate nitrogen (NO_3_^−^-N) was determined using ultraviolet spectrophotometry. Total phosphorus (TP) was measured by molybdenum–antimony anti-colorimetry after digestion with concentrated H_2_SO_4_ and HClO_4_. Available phosphorus (AP) was analyzed using the Olsen-P method. Cation exchange capacity (CEC) was determined by the rapid EDTA-ammonium salt method using a UV-Vis spectrophotometer (METASH UV-9000S, Shanghai, China) [48].

### 2.4. Measurement of N_2_O Emissions

A static dark chamber assembled with a length, width, and height of 50 cm was used for in situ collection of gas. The chamber consists of a box body made from PVC material with a fan for stirring the gas inside and a base made from stainless steel material. A regular grid was established in both KA and NKA to ensure that gas collection points represent the entire area variability. Each chamber was randomly allocateed to each plot in the grid and placed at the center of plot. The gas sample was collected at 9:00–11:00 A.M. while collecting soil samples. The gas was collected in a 60 mL syringe at 0, 10, 20, and 30 min after covering the upper box and then transfered into gas sampling bags. The gas samples were immediately brought back to the laboratory for measurement and analysis through a gas chromatography–mass spectrometry system (Agilent 7890 B, Shanghai, China). N_2_O concentrations were determined using an electron capture detector (ECD) with a detection limit of 1 μg·L^−1^. A standard calibration curve was generated after every 48 samples analyzed. Each gas sample was measured twice. If the relative deviation between the two data is less than 5%, the average value is taken as the final value of the sample. If the deviation exceeds 5%, a third measurement was performed and the average of the two closest data points was used as the final value of the gas sample. Before measuring samples of each batch, a calibration curve containing five concentration points (standard gas of N_2_O at 0.2, 0.5, 1.0, 1.5, 2.0 ppm) was run, and the linear regression coefficient (*R*^2^) of the calibration curve was all ≥0.995. At the beginning and end of each batch of sample measuring, run a high-purity N_2_ (≥99.999%) as a blank to ensure that the entire analysis system was free of contamination. The measured value was only adopted when the *R*^2^ was ≥0.90. The N_2_O emission fluxes (dc/dt) was calculated based on the slope derived from linear regression analysis of four consecutive sample concentration values. The N_2_O emission fluxes was computed using the following formula [49]:

$$F=H\cdot \frac{MP}{R(273+T)}\cdot \frac{dc}{dt}$$

In the formula

*F* represents the gas emission fluxes (mg·m^−2^·h^−1^);
*H* denotes the height of the sampling chamber (m);
*M* is the molar mass of the gas (g·mol^−1^);
*P* indicates the atmospheric pressure at the sampling site (Pa);
*R* is the universal gas constant (8.314 Pa·m^3^·mol^−1^·K^−1^);
*T* represents the average temperature inside the chamber during sampling (°C);
*dc/dt* refers to the gas emission fluxes (μL·L^−1^·min^−1^);

The cumulative N_2_O emission is expressed in kg·hm^−2^.

### 2.5. Calculation of Global Warming Potential

The global warming potential (GWP) was calculated using the following formula [50]:

GWP *= F*_N_2_O_ × 298

where, expressed in terms of CO_2_ emissions, the GWP of a unit mass of N_2_O is 298 times that of CO_2_ [2]. *F*_N_2_O_ denotes the cumulative N_2_O emission (kg·hm^−2^).

### 2.6. Quantification of AOB and nirK-Denitrifierl Abundance

The absolute abundances of AOB and *nirK*-denitrifier were determined using real-time PCR (qPCR). Genomic DNA was extracted using a commercial extraction kit. After assessing purity and concentration, the DNA was used as a template for PCR amplification with barcode-specific primers targeting the selected regions, using TaKaRa Premix Taq^®^ Version 2.0 (TaKaRa Biotechnology Co., Dalian, China). The primer pairs used were as follows: AOB: amoA-F (5′-GGG GTT TCT ACT GGT GGT-3′) and amoA-1R (5′-CCC CTC KGS AAA GCC TTC TTC-3′) [51]; *nirK*-denitrifier: *nirK*-583F (5′-TCA TGG TGC TGC CGC GYG ANG G-3′) and *nirK*-909R (5′-GAA CTT GCC GGT KGC CCA GAC A-3′) [52]. The amplification program consisted of initial denaturation at 95 °C for 3 min, followed by 40 cycles of denaturation at 95 °C for 15 s, and annealing/extension at 60 °C for 30 s. PCR products were verified by agarose gel electrophoresis. The amplification efficiencies for AOB and *nirK*-denitrifier were 95% and 91%, with R^2^ values of 0.933 and 0.928, respectively.

High-throughput sequencing of AOB/*nirK*-denitrifier was conducted by Guangdong Magigene Biotechnology Co., Ltd. (Foshan, China). Each soil sample was sequenced in triplicate, then used for library construction and sequencing. The amplicon libraries were sequenced on the Illumina Nova 6000 platform (San Diego, CA, USA). Data processing was performed using the mothur v.1.48.0 online software. Paired-end reads were merged using FLASH, followed by removal of low-quality bases and adapter-contaminated sequences. ASVs (Amplicon Sequence Variants) were clustered at 97% similarity using the UPARSE algorithm. Representative ASV sequences were selected, and chimeras were removed before data resampling. Taxonomic identification was performed by alignment against the FunGene database.

### 2.7. Data Analysis and Visualization

Initial data processing was performed using Microsoft Excel 2010 (Redmond, WA, USA). Significant differences in soil physicochemical properties, N_2_O emission fluxes, and abundances of AOB/*nirK*-denitrifier between KA and NKA were analyzed by one-way ANOVA-LSD in SPSS 19.0 (IBM Corporation, Amonk, NY, USA). Bar charts of relative bacterial abundances and graphs of N_2_O emission fluxes and cumulative emissions and their correlations were generated using Origin 9.5 (OriginLab Corporation, Northampton, MA, USA). Pearson correlation analysis in SPSS 19.0 (IBM, USA) was conducted to examine relationships between bacterial abundances and N_2_O emission fluxes based on a 95% confidence interval. Bacterial diversity and heat maps illustrating correlations with soil physicochemical factors were computed and plotted using RStudio 2022.07.1+554 (Posit Software, PBC, Boston, MA, USA).

### 2.8. Correlation and Importance Prediction

Partial least squares structural equation modeling (PLS-SEM) and partial least squares regression (PLSR) were performed in RStudio 2024.12.1 to evaluate the correlations and relative importance of soil physicochemical factors, AOB/*nirK*-denitrifiers, and N_2_O emission fluxes in KA and NKA. The 500 bootstrap resampling iterations were used to evaluate the stability of the PLS-SEM model. The robustness of the model parameters was verified by calculating the confidence interval and statistical significance of the path coefficients. At the same time, the composite reliability (CR > 0.7), average variance extraction rate (AVE > 0.5), and cross-validity of the measurement model were evaluated to ensure the reliability of construct measurement.

## 3. Results and Analysis

### 3.1. Soil Factors and Physicochemical Properties

The pH, TP, AP, and exchangeable Ca^2+^ and Mg^2+^ were significantly higher in KA than those in NKA (Table A1). More of sample’s nutrients on eight dates, including SOC, TN, AN, NO_3_^−^-N, and CEC (but NH_4_^+^-N) in KA were significantly higher than that in NKA, indicating a generally higher nutrient level in KA (Table A1). The average values of pH, SOC, TN, NO_3_^−^-N, TP, AP, CEC, exchangeable Ca^2+^, and exchangeable Mg^2+^ across the rice growth in KA remained significantly higher than those in NKA (Table A1). These results demonstrate that KA overall exhibited higher pH, nutrients, and CEC compared to NKA.

### 3.2. N_2_O Emission Fluxes and Cumulative Emissions in KA and NKA

**Figure 1:**
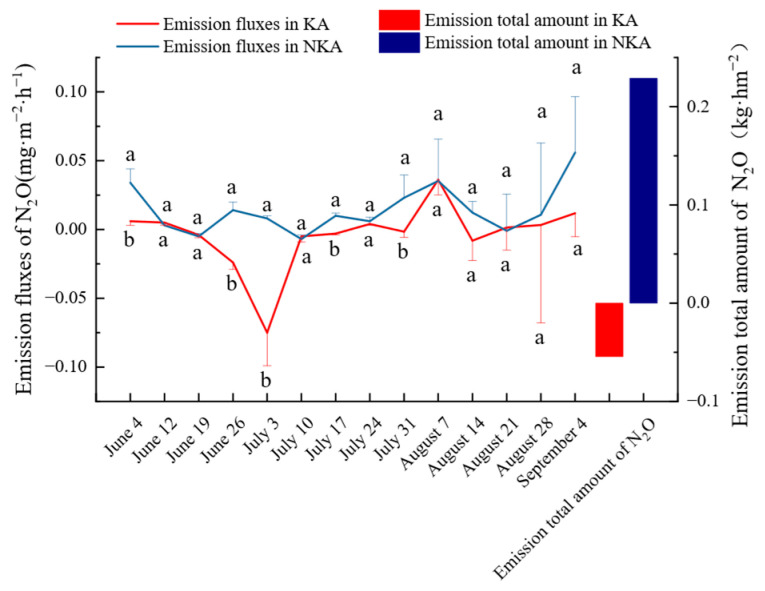
Emission fluxes and total amount of N_2_O from paddy soil in KA and NKA. Note: Different lowercase letters represent significant differences in data at the 0.05 level.

The N_2_O emission fluxes On 4 June (seedling transplanting), 26 June (tillering stage), 3 July (tillering stage), 17 July (jointing stage), and 31 July (booting stage) in KA were significantly lower than those in NKA, while no significant differences were on the other nine dates (Figure 1). The cumulative N_2_O emissions were −0.054 kg·hm^−2^ in KA and 0.229 kg·hm^−2^ in NKA throughout the rice growth period. Thus, the KA soil is a net sink for N_2_O, while the NKA soil is a net source. The global warming potential (GWP) contributed by N_2_O was −16.092 kg·hm^−2^ and 68.242 kg·hm^−2^ for KA and NKA paddy soils, respectively.

### 3.3. Absolute Abundance of AOB/nirK-Denitrifier in KA and NKA

**Figure 2:**
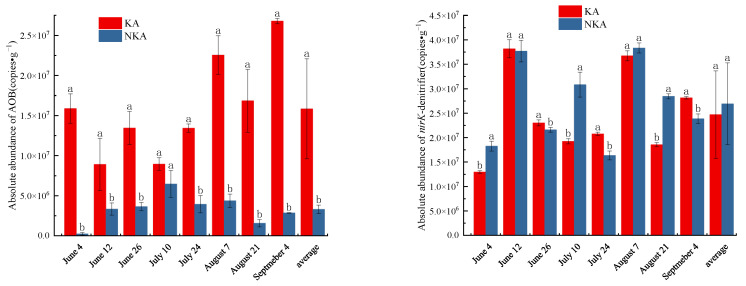
Absolute abundance of AOB and *nirK*-denitrifier in KA and NKA. Note: Different lowercase letters represent significant differences in data at the 0.05 level.

The absolute abundance of AOB was 8.91 × 10^6^–2.68 × 10^7^ copies·g^−1^ in KA, which is higher than that in NKA (1.57 × 10^6^–6.48 × 10^6^ copies·g^−1^), but there was no difference in the absolute abundance of *nirK*-denitrifier in between KA (1.30 × 10^7^–3.82 × 10^7^ copies·g^−1^) and NKA (1.64 × 10^7^–3.84 × 10^7^ copies·g^−1^) (Figure 2).

### 3.4. Community Structure of AOB in KA and NKA

**Figure 3:**
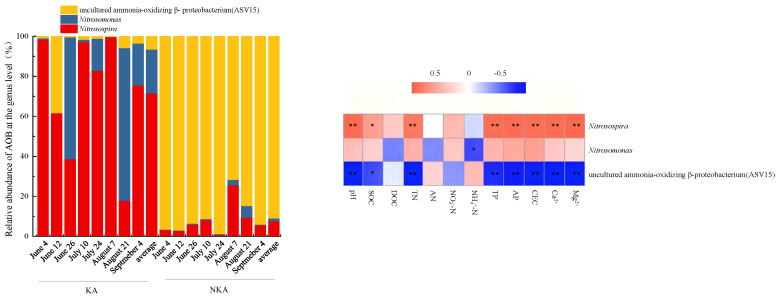
Relative abundance of AOB at the genus level and heat map of the correlation between the abundance of AOB and soil physicochemical factors in KA and NKA. Note: * and ** indicate significant correlations at the 0.05 and 0.01 probability levels, respectively.

The AOB at the genus level in KA was dominated by *Nitrosospira* and *Nitrosomonas*, with the relative abundances of 17.95–98.43% (average 71.48%) and 0–76.28% (average 22.04%), respectively (Figure 3). In NKA, the dominant AOB genus was the uncultured ammonia-oxidizing β-proteobacterium (ASV15), with a relative abundance of 71.62–98.74% and an average of 90.92% (Figure 3). These results indicate significant differences in the composition of AOB between KA and NKA. The relative abundance of *Nitrosospira* showed a significant positive correlation (** *p* < 0.01) with pH, TN, TP, AP, CEC, Mg^2+^, and Ca^2+^, and a significant positive correlation (* *p* < 0.05) with SOC (Figure 3), while the relative abundance of the uncultured ammonia-oxidizing β-proteobacterium (ASV15) was significantly negatively correlated (*p* < 0.05) with the aforementioned soil factors (Figure 3). The relative abundance of *Nitrosomonas* exhibited a significant negative correlation (*p* < 0.05) with NH_4_^+^-N (Figure 3).

The ASVs (Amplicon Sequence Variants) of AOB showed no significant differences between the two areas duiring the rice growth period, and exhibited considerable variability (Table A2).

### 3.5. Community Structure of nirK-Denitrifier in KA and NKA

**Figure 4:**
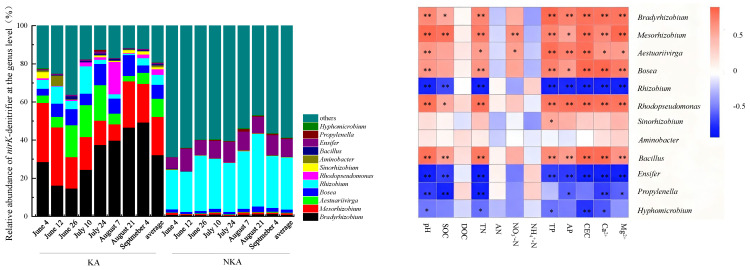
Relative abundance of *nirK*-denitrifier at the genus level and heat map of the correlation between the abundance of *nirK*-denitrifier and soil physicochemical factors in KA and NKA. Note: * and ** indicate significant correlations at the 0.05 and 0.01 probability levels, respectively.

Among the 12 predominance genera, the relative abundances of *Bradyrhizobium*, *Mesorhizobium*, *Aestuariivirga*, and *Bosea* were significantly higher in KA than those in NKA, indicating that the four genera are typical *nirK*-denitrifier in KA (Figure 4). All four belong to Hyphomicrobiales (order), and are classified into four families, Bradyrhizobiaceae, Phyllobacteriaceae, Aestuariivirgaceae, and Boseaceae, respectively. In contrast, the relative abundances of *Rhizobium* and *Ensifer* were significantly higher in NKA, identifying them as unique denitrifying genera in NKA (Figure 4). Both genera belong to the family of Rhizobiaceae within Hyphomicrobiales (order). These results demonstrate significant differences in *nirK*-denitrifier between KA and NKA. As also shown in Figure 4, the relative abundances of *Bradyrhizobium*, *Mesorhizobium*, *Aestuariivirga*, *Bosea*, *Rhodopseudomonas*, and *Bacillus* were significantly positively correlated with physicochemical factors including pH, Mg^2+^, Ca^2+^, CEC, TN, TP, AP, SOC (but *Aestuariivirga* and *Bosea*), and NO_3_^−^-N (but *Bradyrhizobium*, *Bosea*, *Bacillus*, and *Rhodopseudomonas*). Conversely, the relative abundances of four genera (*Rhizobium*, *Ensifer*, *Propylenella*, and *Hyphomicrobium*) were significant negative correlations with pH, SOC (but *Hyphomicrobium*), TN, TP (but *Propylenella*), AP (but *Hyphomicrobium*), CEC (but *Propylenella*), Ca^2+^, and Mg^2+^ (but *Hyphomicrobium*).

**Figure A1:**
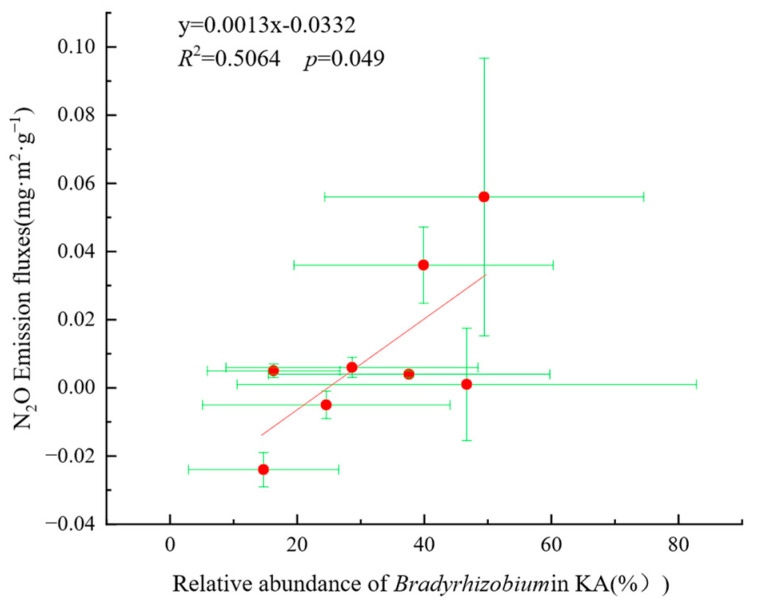
Correlation between relative abundance of *Bradyrhizobium* and N_2_O emission fluxes in KA. Note: the red dots represent scatter points, and the green lines represent error bars.

The significant positive correlation between the relative abundance of *Bradyrhizobium* and N_2_O emission fluxes (Figure A1) indicates that *Bradyrhizobium* may have a considerable influence on N_2_O emissions.

The Chao1 index showed no significant differences between KA and NKA throughout rice growth period, while the number of ASVs in KA was significantly higher than that in NKA at all stages (Table A3). Additionally, the Shannon and Simpson indices in KA before 21 August (heading) were significantly higher than those in NKA (Table A3). These results demonstrate significant diversity differences in *nirK*-denitrifier between the two areas.

The correlation analysis between diversity indices of *nirK*-denitrifier and soil physicochemical factors is summarized in Table 1. The number of ASVs were significant positive correlations with pH, SOC, TN, NO_3_^−^-N, TP, AP, CEC, Ca^2+^, and Mg^2+^. The Chao1 index was significantly positively correlated with pH, NO_3_^−^-N, CEC, and Mg^2+^. The ACE index exhibited significant positive correlations with pH, SOC, TN, CEC, and Mg^2+^. Both the Shannon and Simpson indices were significantly positively correlated with pH, SOC, NO_3_^−^-N, TP, AP, CEC, Ca^2+^, and Mg^2+^. These correlations indicate that the diversity of *nirK*-denitrifier in both KA and NKA is regulated by multiple key physicochemical factors, including pH, NO_3_^−^-N, SOC, Ca^2+^, Mg^2+^, and CEC. Furthermore, the distinct soil environment of KA supports a unique *nirK*-denitrifier community with higher diversity.

### 3.6. Correlation and Relative Importance of Soil Physicochemical Factors, AOB, nirK-Denitrifier, and N_2_O Emission Fluxes in KA and NKA

**Figure 5:**
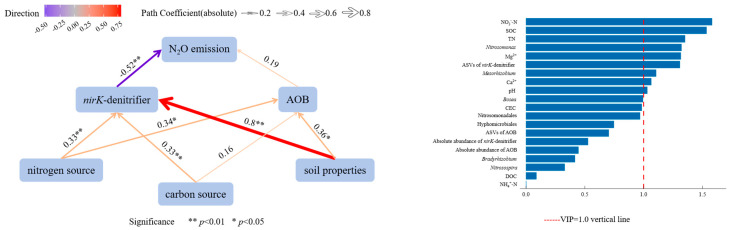
The correlation and significance of soil physicochemical factors, AOB, *nirK*-denitrifier, and N_2_O emission fluxes in KA and NKA. Note: soil properties include pH, Ca^2+^, Mg^2+^ and CEC; carbon source includes SOC and DOC; nitrogen source includes TN, NH_4_^+^-N, and NO_3_^−^-N; AOB includes ASVs, absolute abundance, *Nitrosospira*, *Nitrosomonas,* and uncultured ammonia-oxidizing β-proteobacterium (ASV15); *nirK*-denitrifier includes ASVs, absolute abundance, Hyphomicrobiales, *Bradyrhizobium*, and *Bosea*.

The results as predicted by PLS-SEM and PLSR indicate that soil properties, carbon sources, and nitrogen sources exerted positive effects on both AOB and *nirK*-denitrifier (Figure 5). The sensitivity analysis of the PLS-SEM model showed that all critical path coefficients remained statistically significant (*p* < 0.05) in Bootstrap validation. The measurement model showed good reliability (CR > 0.7), convergent validity (AVE > 0.5), and acceptability (GoF = 0.67 > 0.1), confirming the robustness of the model results. AOB showed a positive correlation with N_2_O emission fluxes, whereas *nirK*-denitrifier exhibited a significant negative correlation with N_2_O emission fluxes (Figure 5). These correlations demonstrate that N_2_O emission fluxes is directly influenced by both AOB and *nirK*-denitrifier.

Environmental factors identified as having considerable importance for N_2_O emission fluxes included NO_3_^−^-N, SOC, TN, Mg^2+^, Ca^2+^, and pH. Key microbial factors included *Nitrosomonas*, ASVs of *nirK*-denitrifier, and *Mesorhizobium* (Figure 5). This indicates that both environmental factors and microbes significantly influence N_2_O emissions.

## 4. Discussion

### 4.1. Differences in N_2_O Emissions Between KA and NKA and Their Environmental Drivers

Meta-analyses of global data suggest that environmental factors such as soil carbon and nitrogen content, pH, and clay minerals are primary regulators of the relative contributions of nitrification/denitrification ratio of N_2_O emissions [21,53]. In this study, KA with higher pH exhibited significantly lower N_2_O emission fluxes and cumulative emissions compared to NKA. To our knowledge, this is the first report of low N_2_O emissions from soil in KA during rice growth. This finding aligns with reports that N_2_O emissions are more pronounced under aerobic conditions with high NO_3_^−^-N [54], and that the consumption of NH_4_^+^-N and accumulation of NO_3_^−^-N are positively correlated with N_2_O emissions in paddy soils [55]. Furthermore, incubation experiments using ^15^N tracing on ten soils from southern China with varying pH and SOC also demonstrated that increasing soil pH reduces N_2_O emissions [56]. Elevated soil pH promotes the conversion of NO_2_^−^ to NO_3_^−^, thereby decreasing the concentrations of NH_2_OH and NO_2_^−^ (key precursors of N_2_O formation) and ultimately mitigating N_2_O emissions [57]. This is consistent with the higher NO_3_^−^-N observed in KA in our study. Therefore, higher content of NO_3_^−^-N and pH in could be an important reason of lower N_2_O emissions in KA. Similarly, amending acidic soils with biochar to raise pH and increase NO_3_^−^-N content could effectively reduce N_2_O emissions [18].

SOC is also another major factor in regulating denitrification potential [58]. Labile carbon continuously supplied from the rhizosphere profoundly influences terrestrial nitrogen cycling [56], which may be utilized by *nirK*-denitrifier, enhancing denitrification activity and potentially increasing N_2_O emissions. In our study, carbon source was identified as the second-most important factor affecting N_2_O emissions. A possible explanation is that Ca^2+^ binds with DOC and humic acids to form insoluble calcium humate, altering the availability of carbon to *nirK*-denitrifier and thus influencing N_2_O emission processes [59]. At the same time, nitrogen elements in karst soil are partly stabilized due to their binding with calcium minerals, which also limits the nitrogen utilization by *nirK*-denitrifier. In farmland, fertilization promotes the accumulation of organic matter, which stimulates denitrifying bacteria to reduce N_2_O to N_2_, leading to net absorption of N_2_O [16]. Wang et al. [60] reported that applying alkaline amendments such as lime can also reduce N_2_O emissions in acidic soils (pH < 5.5). Hence, in this study, the lower N_2_O emissions in KA are associated with their alkaline environment and high Ca^2+^ content. In agricultural production, soil amendments such as adding lime, SOC, and adjusting pH (such as biochar) can be used to reduce N loss and N_2_O emissions in acidic soils.

The Mg^2+^ content in KA was significantly higher than that in NKA. Mg^2+^ showed a significant negative correlation with the Shannon and Simpson indices of *nirK*-denitrifier, and PLS-SEM predictions indicated that Mg^2+^ significantly influences *nirK*-denitrifier, suggesting a key role for Mg^2+^ in shaping *nirK*-denitrifier assemblages. Studies have shown that Mg-modified biochar (BCMg) reduces N_2_O emissions by increasing pH and decreasing the activity of N_2_O-producing bacteria in coastal saline soils [61]. Additionally, short-term incubation experiments demonstrated that Mg-fertilizer can mitigate N_2_O production in agricultural soils [62]. These findings imply that Mg^2+^ may influence N_2_O emissions by altering the community structure and function of *nirK*-denitrifier. The mechanism by which Mg affects *nirK*-denitrifier to reduce N_2_O emissions in KA deserves further investigation. The above research also suggests that we can reduce N_2_O emissions by applying Mg-fertilizer in NKA.

Meta-analyses indicate that increased N-fertilizer application significantly enhances soil N_2_O emissions [57,63], though native soil nitrogen (rather than fertilizer-derived nitrogen) is the main contributor to N_2_O emissions, accounting for 67% of total emissions [56]. In upland soils of the North China Plain, a significant linear relationship exists between the available nitrogen and N_2_O emissions (*p* < 0.01) [64]. In our study, nitrogen sources influenced N_2_O emissions by affecting *nirK*-denitrifier. KA had higher TN and NO_3_^−^-N, while the NKA had higher NH_4_^+^-N. NO_3_^−^-N and TN were among the most important factors affecting N_2_O emissions. High NO_3_^−^-N content provides substrate for AOB while also reducing N_2_O emissions [57]. Thus, N_2_O emissions in KA may be regulated by NO_3_^−^-N dynamics, suggesting different soil N_2_O emission mechanisms between KA and NKA. Future studies could use ^15^N stable isotope tracing to test this hypothesis. In summary, soil factors indirectly drive N_2_O emissions by influencing the core processes of nitrification and denitrification [12]. And Ca^2+^ may combine with organic nitrogen to affect the nitrogen availability and change the concentration of NO_3_^−^-N and NH_4_^+^-N, thus affecting the activities of AOB and *nirK*-denitrifier. Understanding N_2_O emissions therefore requires integrating soil environmental factors with microbial processes.

### 4.2. Dominant AOB Communities in KA and Their Underlying Causes

Microbially mediated nitrification and denitrification are core processes governing N_2_O emissions in paddy soils [12,65]. In this study, the absolute abundance of AOB was significantly higher in KA than in NKA. Specifically, the relative abundance of *Nitrosospira* (71.48%) was significantly higher in KA compared to NKA (7.71%). Ke et al. [66] reported that *Nitrosospira* prefers low NH_4_^+^-N environments, which aligns with our finding of higher relative abundance of *Nitrosospira* in the low NH_4_^+^-N in KA than the high NH_4_^+^-N in NKA. This is further supported by studies showing a significant positive correlation between TN and the relative abundance of *Nitrosospira* [67], and that N-fertilization enhances the competitive advantage of *Nitrosospira* Cluster 3 [68]. In summary, the neutral alkaline KA had a significantly higher relative abundance of *Nitrosospira* than the acidic NKA, indicating that *Nitrosospira* is a typical and dominant nitrifier in KA, representing a key distinction from NKA.

*Nitrosomonas* was the second dominant genus in KA, with an average relative abundance of 22.04% (compared to 1.37% in NKA). Studies have identified *Nitrosomonas* as the third dominant genus in KA soils under four vegetations [69]. The relative abundance of *Nitrosomonas* was significantly negatively correlated with NH_4_^+^-N (Figure 3), suggesting that the low NH_4_^+^-N content in KA may be a limiting factor for this genus. Research indicates that the ammonia oxidation rate of *N. europaea* (a common species of *Nitrosomonas*) peaks at pH 6.7–7.0 and is not strictly dependent on NH_4_^+^-N availability [70], a condition consistent with the neutral alkaline pH and low NH_4_^+^-N environment of KA in this study.

Aggregate size significantly influences the relative abundance of *Nitrosospira* [71]. Huang et al. [72] found that *Nitrosospira* is primarily concentrated in micro-aggregates, whereas Jiang et al. [73] reported a higher proportion of *Nitrosospira* in macro-aggregates. Additionally, Hou et al. [74] noted that *Nitrosomonas* is relatively more abundant in micro-aggregates. Future research should therefore explore the niche differentiation of AOB populations in aggregates and their underlying mechanisms in KA and NKA.

### 4.3. Relationship Between AOB and N_2_O Emissions

**Figure A2:**
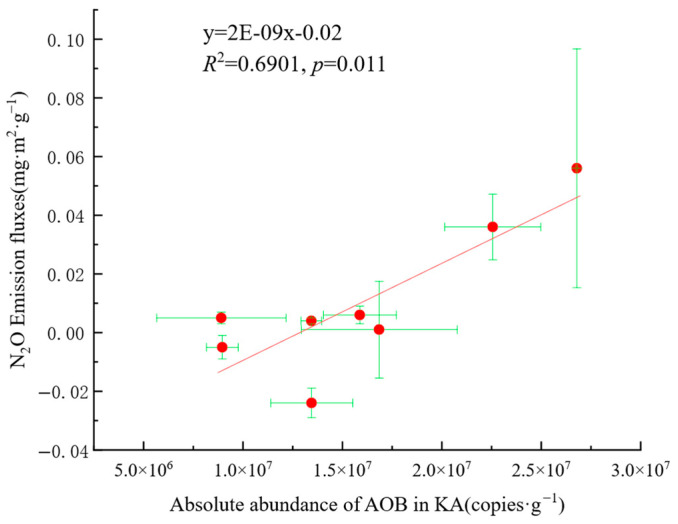
Correlation between absolute abundance of AOB and N_2_O emission fluxes in KA. Note: the red dots represent scatter points, and the green lines represent error bars.

From a global perspective, nitrification contributes more to N_2_O emissions on average than denitrification, though the relative contributions vary across terrestrial ecosystems [21]. In this study, the absolute abundance of AOB was significantly higher in KA and positively correlated with N_2_O emission fluxes (Figure A2), suggesting that AOB may substantially contribute to N_2_O emissions in KA paddy soils. Similarly, in KA forest soils of Guizhou, China, autotrophic nitrification pathways (ammonia oxidation, nitrifier denitrification, and nitrification-coupled denitrification) account for over 70% of total N_2_O emission [75]; the abundance of AOB, organic nitrogen mineralization, and total autotrophic nitrification rates are positively correlated with N_2_O emissions [10]. Thus, nitrification is likely the dominant process driving N_2_O emissions in KA. In this study, *Nitrosomonas* had a high importance value for N_2_O emissions, indicating a potential intrinsic link between its relative abundance and N_2_O emissions. However, studies show that *Nitrosomonas* europaea can perform denitrification using NH_4_^+^ or H_2_ as electron donors and ON_2_^−^ as an electron acceptor, producing N_2_O and leading to N loss [76,77]. Notably, *N*. *europaea* produces N_2_O only under O_2_-limited conditions [76]. Other reports indicate that *N. eutropha* can utilize N_2_O in anaerobic ammonia oxidation [78]. These findings highlight the functional diversity of *Nitrosomonas*, which complicates its contribution to N_2_O emissions. Environmental factors such as pH, O_2_, and NH_4_^+^-N likely play decisive roles in driving the nitrifying/denitrifying functions of this genus. Future research should focus on the functional diversity of *Nitrosomonas* in KA and NKA and its actual contribution to N_2_O emissions.

Meta-analyses suggest that the relative abundance ratio of AOB and ammonia-oxidizing archaea to denitrifiers is a key driver of N_2_O emissions [21]. However, another meta-analysis of 101 global field monitoring datasets showed no correlation between soil N_2_O emissions and AOB abundance [64]. These contrasting results may imply that interactions between AOB and other microbia in specific ecosystems are also critical in determining N_2_O emission contributions. Therefore, clarifying the assembly mechanisms of specific microbial communities and identifying key ecological factors influencing N_2_O emissions in KA are particularly important.

### 4.4. Differences in nirK-Denitrifier Community Structure Between KA and NKA and Their Causes

Meta-analyses of global data indicate that N-fertilizer application significantly increases the absolute abundance of *nirK*-denitrifiers in agricultural soils [57,58], primarily because N-fertilizers provide a sufficient substrate for them.

In this study, the diversities of *nirK*-denitrifier differed significantly between KA and NKA (Table A3). Moreover, ASVs of *nirK*-denitrifier and multiple diversity indices correlated with various soil physicochemical factors, including pH, SOC, TN, NO_3_^−^-N, Ca^2+^, and Mg^2+^ (Table 1), indicating that the distinct soil environment of KA supports a unique and more diverse *nirK*-denitrifier. No correlation was between the absolute abundance of *nirK*-denitrifiers and soil physicochemical factors in either area or overall. However, PLS-SEM predictions showed that soil properties, nitrogen sources, carbon sources, and phosphorus sources positively affect *nirK*-denitrifier (Figure 5), suggesting that *nirK*-denitrifiers are regulated by multiple interconnected environmental factors rather than single factor [79].

In KA, *Bradyrhizobium* was the most abundant *nirK*-type denitrifier and showed a significant positive correlation with N_2_O emission fluxes (Figure A1). Studies indicate that *Bradyrhizobium* possesses N-fixing [80] and P-solubilizing abilities [81]. *Bradyrhizobium* is sensitive to warming, which reduces its relative abundance, while N-fertilizer increases its relative abundance under warmed conditions [82]. *Bradyrhizobium* has also been reported as a *nirS*-type denitrifier [83]. These results highlight the genetic and functional diversity of *Bradyrhizobium*, suggesting that its ecological role may contribute significantly to N_2_O emissions in KA. *Aestuariivirga* was the third-most dominant denitrifying genus in KA. Reportedly, *Aestuariivirga* litoralis shares high DNA sequence similarity with bacteria of Rhizobiales [84], implying that *Aestuariivirga* may also have N-fixing capabilities.

### 4.5. Relationship Between nirK-Denitrifiers and N_2_O Emissions

In this study, ASVs of *nirK*-denitrifier and *Mesorhizobium* had high importance values for N_2_O emissions, and *nirK*-denitrifier overall had a negative effect on N_2_O emissions, suggesting that *Mesorhizobium* may contribute little to N_2_O emissions. *Mesorhizobium* was the second dominant denitrifier in KA, with a significantly higher relative abundance than in NKA. Research shows that *Mesorhizobium* also has N-fixing [85] and P-solubilizing abilities [81], and increasing NH_4_^+^-N can enhance its abundance [81]. In this study, NH_4_^+^-N was generally low in KA, so NH_4_^+^-N fertilizer might increase the relative abundance of *Mesorhizobium*. Some strains of *Mesorhizobium* harbor N_2_O reductase (*nosZ*), which reduces N_2_O to N_2_ and thus reduces N_2_O emissions [86]. Moreover, the reduction in N_2_O by *Mesorizobium* may be enhanced by the neutral alkaline pH in KA, resulting in negative N_2_O emissions. These results indicate that the unique neutral alkaline and Ca-rich environment of KA supports *nirK*-denitrifiers with diverse N metabolic functions, resulting in negative N_2_O emissions. However, whether *Mesorhizobium* can truly reduce N_2_O emissions in KA still needs to be verified by adding different *Mesorhizobium* strains. Recently, studies using isotope tracing show that fungal-mediated denitrification is the largest contributor of N_2_O emissions (51–63%) compared to bacterial and chemical denitrification in paddy soils [54]. Therefore, future research should investigate the contributions of fungi to N_2_O emissions in KA and NKA [16].

In summary, the possible reasons for the net absorption of N_2_O in KA is that the alkaline and Ca-rich environment suppress the production of N_2_O and a portion of N_2_O is reduced by microorganisms. The above findings suggest that we can reduce soil N_2_O emissions by adding lime, Mg-fertilizer, SOC, NO_3_^−^-N, pH adjusters, and specific microorganisms in NKA paddy soils, which is important for agricultural emission reduction practices under global climate change.

## 5. Conclusions

(1) The cumulative N_2_O emissions were −0.054 kg·hm^−2^ in KA and 0.229 kg·hm^−2^ in the NKA throughout the rice growth period, respectively, indicating that karst rice fields are reservoirs of N_2_O.
(2) The absolute abundance of AOB was significantly higher in KA than that in NKA, whereas the absolute abundance of *nirK*-denitrifier did not differ significantly between the two areas.
(3) The dominant AOB in KA were *Nitrosospira* and *Nitrosomonas*, while the dominant AOB in NKA was an uncultured ammonia-oxidizing β-proteobacterium. The dominant *nirK*-denitrifiers in KA were *Bradyrhizobium*, *Mesorhizobium*, *Aestuariivirga*, and *Bosea*, whereas *Rhizobium* and *Ensifer* were dominant in NKA.
(4) Soil properties, nitrogen sources, and carbon sources had positive effects on AOB, while soil properties, nitrogen sources, and phosphorus sources positively affected AOB. The *nirK*-denitrifiers had a negative effect on N_2_O emission fluxes. Environmental factors with high importance for N_2_O emission fluxes included NO_3_^−^-N, SOC, TN, Mg^2+^, Ca^2+^, and pH, and key microbial factors were *Nitrosomonas*, ASVs of *nirK*-denitrifiers, and *Mesorhizobium*, indicating that these factors significantly influence N_2_O emissions.

## Figures and Tables

**Table 1:** Correlation between diversity index of *nirK*-denitrifier and soil physcicochemical factors.

Diversity	pH	SOC	DOC	TN	AN	NH_4_^+^-N	NO_3_^−^-N	TP	AP	CEC	Ca^2+^	Mg^2+^
ASVs	0.915 **	0.749 **	−0.015	0.773 **	−0.168	−0.187	0.551 *	0.725 **	0.723 **	0.935 **	0.739 **	0.879 **
Chao1 Index	0.466 *	0.371	−0.044	0.405	−0.193	−0.161	0.518 *	0.189	0.168	0.496 *	0.249	0.506 *
ACE Index	0.522 *	0.466 *	−0.144	0.600 **	−0.197	−0.19	0.405	0.194	0.379	0.554 *	0.314	0.569 *
Shannon Index	−0.793 **	−0.568 *	−0.038	−0.389	0.25	0.209	−0.648 **	−0.792 **	−0.559 *	−0.837 **	−0.634 **	−0.733 **
Simpson Index	−0.746 **	−0.500 *	−0.036	−0.318	0.27	0.215	−0.632 **	−0.783 **	−0.514 *	−0.784 **	−0.555 *	−0.689 **

Note: * and ** indicate significant correlations at the 0.05 and 0.01 probability levels, respectively. Abbreviations: SOC: soil organic carbon; DOC: dissolved organic carbon; TN: total nitrogen; AN: available nitrogen; NH_4_^+^-N: ammonium nitrogen; NO_3_^−^-N: nitrate nitrogen; TP: total phosphorus; AP: available phosphorus; CEC: cation exchange capacity; Ca^2+^: exchangeable calcium ion; Mg^2+^: exchangeable magnesium ion.

## Data Availability

The original contributions presented in this study are included in the article. Further inquiries can be directed to the corresponding author.

## Appendix A

**Table A1:** Characteristics of soil physicochemical factors in KA and NKA during the rice growing.

	Areas	4 June	12 June	26 June	10 July	24 July	7 August	21 August	4 September	Average
pH	KA	7.50 ± 0.04 a	7.26 ± 0.61 a	7.39 ± 0.13 a	7.57 ± 0.29 a	7.09 ± 0.11 a	7.38 ± 0.14 a	7.50 ± 0.21 a	7.31 ± 0.25 a	7.38 ± 0.16 a
	NKA	5.01 ± 0.26 b	4.90 ± 0.23 b	6.23 ± 0.5 b	4.82 ± 0.11 b	4.78 ± 0.06 b	4.88 ± 0.27 b	4.74 ± 0.14 b	4.80 ± 0.31 b	5.02 ± 0.50 b
SOC (g/kg)	KA	15.42 ± 0.37 a	14.43 ± 1.10 a	14.79 ± 0.90 a	14.52 ± 1.72 a	13.27 ± 1.76 a	13.48 ± 0.42 a	14.11 ± 0.95 a	14.48 ± 2.09 a	14.31 ± 0.69 a
	NKA	13.78 ± 1.01 b	14.05 ± 1.27 a	12.07 ± 1.56 b	12.59 ± 0.37 b	10.19 ± 1.42 b	10.01 ± 0.94 b	11.14 ± 0.73 b	9.74 ± 3.39 b	11.70 ± 1.69 b
DOC (mg/kg)	KA	0.23 ± 0.03 b	1.06 ± 0.1 a	0.21 ± 0.04 b	0.70 ± 0.02 a	0.90 ± 0.13 a	0.56 ± 0.09 a	0.24 ± 0.10 a	0.25 ± 0.06 a	0.52 ± 0.34 a
	NKA	0.38 ± 0.07 a	0.32 ± 0.08 b	1.65 ± 0.15 a	0.38 ± 0.03 b	0.21 ± 0.06 b	0.44 ± 0.21 a	0.17 ± 0.04 a	0.26 ± 0.09 a	0.48 ± 0.48 a
TN (g/kg)	KA	1.90 ± 0.03 a	1.71 ± 0.03 a	1.91 ± 0.05 a	1.84 ± 0.03 a	1.69 ± 0.03 a	1.91 ± 0.31 a	1.97 ± 0.00 a	1.91 ± 0.10 a	1.86 ± 0.10 a
	NKA	1.68 ± 0.03 b	1.57 ± 0.02 b	1.72 ± 0.03 b	1.67 ± 0.06 b	1.52 ± 0.14 a	1.55 ± 0.04 a	1.36 ± 0.04 b	1.17 ± 0.02 b	1.53 ± 0.19 b
AN (mg/kg)	KA	55.49 ± 5.67 b	91.43 ± 2.04 a	56.43 ± 10.30 b	98.89 ± 2.13 a	91.48 ± 9.15 a	83.96 ± 18.16 a	64.58 ± 2.01 a	69.03 ± 3.83 a	76.41 ± 17.10 a
	NKA	93.25 ± 8.11 a	92.57 ± 1.47 a	158.05 ± 7.10 a	78.57 ± 5.33 b	65.33 ± 1.58 b	71.60 ± 7.16 a	56.47 ± 2.01 b	53.53 ± 1.76 b	83.67 ± 33.51 a
NH_4_^+^-N (mg/kg)	KA	11.55 ± 0.37 b	32.41 ± 0.15 b	13.61 ± 0.31 b	27.82 ± 1.38 b	26.5 ± 0.45 b	22.2 ± 0.17 b	13.76 ± 0.45 b	13.81 ± 0.15 b	20.21 ± 8.03 b
	NKA	46.67 ± 0.60 a	55.18 ± 1.32 a	88.60 ± 4.00 a	50.56 ± 0.55 b	47.63 ± 0.27 b	32.21 ± 0.32 a	25.76 ± 0.44 a	24.77 ± 0.36 a	40.40 ± 12.51 a
NO_3_^−^-N (mg/kg)	KA	39.30 ± 0.77 a	54.29 ± 10.65 a	45.64 ± 1.11 a	49.96 ± 6.95 a	35.76 ± 13.72 a	26.43 ± 2.33 a	36.44 ± 0.41 a	40.79 ± 0.17 a	41.08 ± 8.80 a
	NKA	25.99 ± 1.18 b	22.87 ± 1.09 b	8.83 ± 0.62 b	27.49 ± 1.79 b	30.25 ± 1.64 b	9.61 ± 0.95 b	31.77 ± 0.82 b	29.02 ± 0.52 b	23.23 ± 9.06 b
TP (g/kg)	KA	0.32 ± 0.08 a	0.21 ± 0.01 a	0.15 ± 0.03 a	0.18 ± 0.03 a	0.33 ± 0.04 a	0.21 ± 0.04 a	0.24 ± 0.03 a	0.26 ± 0.03 a	0.24 ± 0.06 a
	NKA	0.08 ± 0.04 b	0.14 ± 0.01 b	0.07 ± 0.01 b	0.08 ± 0.02 b	0.13 ± 0.04 b	0.11 ± 0.02 b	0.12 ± 0.01 b	0.13 ± 0.02 b	0.11 ± 0.03 b
AP (mg/kg)	KA	30.53 ± 3.09 a	12.82 ± 1.05 a	27.76 ± 1.53 a	20.09 ± 0.17 a	24.14 ± 2.46 a	42.38 ± 6.79 a	23.47 ± 1.87 a	33.73 ± 6.10 a	26.87 ± 8.98 a
	NKA	13.05 ± 1.53 b	7.60 ± 0.55 b	15.99 ± 0.72 b	15.89 ± 0.24 b	14.60 ± 0.10 b	12.88 ± 3.44 b	11.60 ± 3.78 b	12.59 ± 1.04 b	13.03 ± 2.70 b
CEC (cmol/kg)	KA	6.51 ± 2.40 a	6.47 ± 1.25 a	6.97 ± 1.39 a	7.30 ± 0.77 a	7.87 ± 1.29 a	6.86 ± 0.99 a	8.34 ± 0.18 a	6.98 ± 2.49 a	7.16 ± 0.65 a
	NKA	4.14 ± 0.25 a	3.97 ± 0.35 b	4.58 ± 0.09 b	3.92 ± 0.82 b	3.55 ± 0.66 b	4.05 ± 0.27 b	2.84 ± 0.67 b	3.58 ± 0.13 a	3.83 ± 0.51 b
Ca^2+ (^cmol/kg)	KA	4.83 ± 0.16 a	4.73 ± 0.33 a	5.02 ± 0.14 a	4.63 ± 0.19 a	4.89 ± 0.25 a	5.08 ± 0.27 a	4.96 ± 0.17 a	4.64 ± 0.29 a	4.85 ± 0.17 a
	NKA	2.43 ± 0.22 b	2.44 ± 0.3 b	2.41 ± 0.03 b	2.30 ± 0.01 b	1.87 ± 0.32 b	2.20 ± 0.16 b	2.34 ± 0.22 b	2.08 ± 0.03 b	2.26 ± 0.20 b
Mg^2+ (^cmol/kg)	KA	0.26 ± 0.02 a	0.24 ± 0.01 a	0.23 ± 0.00 a	0.23 ± 0.01 a	0.24 ± 0.00 a	0.24 ± 0.00 a	0.24 ± 0.00 a	0.23 ± 0.01 a	0.24 ± 0.01 a
	NKA	0.21 ± 0.00 b	0.22 ± 0.00 b	0.22 ± 0.00 b	0.21 ± 0.01 b	0.15 ± 0.02 b	0.17 ± 0.00 b	0.20 ± 0.01 b	0.16 ± 0.00 b	0.19 ± 0.03 b

Note: Values in the table represent (mean ± standard deviation). Lowercase letters following numbers indicate significant differences between KA and NKA on the same sampling date at the 0.05 probability level. Abbreviations: SOC, soil organic carbon; DOC, dissolved organic carbon; TN, total nitrogen; AN, available nitrogen; NH_4_^+^-N, ammonium nitrogen; NO_3_^−^-N, nitrate nitrogen; TP, total phosphorus; AP, available phosphorus; CEC, cation exchange capacity; Ca^2+^, exchangeable calcium ion; Mg^2+^, exchangeable magnesium ion.

**Table A2:** Diversity index of AOB in KA and NKA.

Diversity	Areas	4 June	12 June	26 June	10 July	24 July	7 August	21 August	4 September	Average
ASVs	KA	268 ± 134 a	536 ± 592 a	268 ± 101 a	416 ± 424 a	475 ± 233 a	25 ± 23 a	28 ± 19 a	78 ± 43 a	262 ± 203 a
	NKA	212 ± 356 a	177 ± 200 a	936 ± 40 a	83 ± 61 a	288 ± 270 a	2039 ± 3243 a	52 ± 26 a	132 ± 153 a	490 ± 687 a

Note: Values in the table represent (mean ± standard deviation). Lowercase letters following numbers indicate significant differences between KA and NKA on the same sampling date at the 0.05 probability level.

**Table A3:** Diversity indices of *nirK*-denitrifier in KA and NKA.

Diversity	Areas	4 June	12 June	26 June	10 July	24 July	7 August	21 August	4 September	Average
Chao1 Index	KA	9661 ± 2658 a	5916 ± 1475 a	9990 ± 2730 a	8417 ± 2614 a	6964 ± 2732 a	7501 ± 1210 a	6999 ± 2269 a	5462 ± 2007 a	7614 ± 1639 a
	NKA	1988 ± 1230 b	2461 ± 2050 a	2105 ± 864 b	1642 ± 550 b	2176 ± 371 b	2207 ± 1117 b	1447 ± 583 b	1318 ± 695 b	1918 ± 404 b
ACE Index	KA	43.27 ± 5.72 a	70.01 ± 52.05 a	68.04 ± 16.88 a	42.88 ± 6.04 a	42.80 ± 4.93 a	48.56 ± 7.51 a	70.48 ± 32.23 a	46.05 ± 17.83 a	54.01 ± 12.99 a
	NKA	52.67 ± 17.47 a	40.06 ±1.42 a	40.46 ± 4.46 a	39.31 ± 8.00 a	49.44 ± 22.15 a	41.92 ± 6.42 a	34.87 ± 9.42 a	47.50 ± 30.64 a	43.28 ± 5.98 a
Shannon Index	KA	47.19 ± 2.85 a	53.98 ±16.25 a	87.36 ± 24.70 a	47.77 ± 9.54 a	50.68 ± 3.65 a	50.77 ± 5.37 a	66.42 ± 23.25 a	50.28 ± 21.76 a	56.81 ± 13.76 a
	NKA	51.21 ± 11.81 a	43.77 ± 3.16 a	43.86 ± 5.34 b	43.65 ± 7.80 a	53.52 ± 24.72 a	45.06 ± 5.97 a	35.45 ± 10.42 a	37.72 ± 13.76 a	44.28 ± 6.05 a
Simpson Index	KA	2.19 ± 0.20 a	2.36 ± 0.19 a	2.29 ± 0.15 a	2.41 ± 0.07 a	2.40 ± 0.47 a	2.46 ± 0.23 a	1.95 ±0.12 a	1.73 ± 0.69 a	2.22 ± 0.26 a
	NKA	1.32 ± 0.10 b	1.41 ± 0.29 b	1.58 ±0.12 b	1.61 ± 0.18 b	1.41 ± 0.43 b	1.70 ± 0.18 b	1.79 ±0.12 a	1.68 ± 0.43 a	1.56 + 0.17 b
Chao1 Index	KA	0.79 ±0.05 a	0.85 ± 0.04 a	0.82 ± 0.04 a	0.87 ± 0.01 a	0.83 ± 0.08 a	0.86 ± 0.04 a	0.74 ± 0.06 a	0.62 ±0.25 a	0.80 ± 0.08 a
	NKA	0.52 ± 0.05 b	0.56 ± 0.12 b	0.61 ± 0.03 b	0.63 ± 0.06 b	0.55 ± 0.19 b	0.67 ± 0.03 b	0.69 ± 0.02 a	0.65 ± 0.11 a	0.61 ± 0.06 b

Note: Values in the table represent (mean ± standard deviation). Lowercase letters following numbers indicate significant differences between KA and NKA on the same sampling date at the 0.05 probability level.
